# Whole-Genome Shotgun Sequences of *Salmonella enterica* Serovar Typhimurium Lilleengen Type Strains LT1, LT18, LT19, LT20, LT21, and LT22

**DOI:** 10.1128/genomeA.00720-17

**Published:** 2017-07-27

**Authors:** Robert A. Kazmierczak, Aaron A. Best, Duy Nguyen, Abraham Eisenstark

**Affiliations:** a Cancer Research Center, Columbia, Missouri, USA; b Division of Biological Sciences, University of Missouri, Columbia, Missouri, USA; c Department of Biology, Hope College, Holland, Michigan, USA

## Abstract

The Lilleengen type (LT) collection of *Salmonella enterica* serovar Typhimurium strains has served the scientific community as a group of model organisms for basic genetic and biochemical pathway research. Here, we report the whole-genome shotgun sequences of *Salmonella enterica* serovar Typhimurium strains LT1, LT18, LT19, LT20, LT21, and LT22.

## GENOME ANNOUNCEMENT

The Lilleengen type (LT) collection of *Salmonella enterica* serovar Typhimurium strains has served the scientific community as a group of model organisms, including those for basic genetic and biochemical pathway research (LT2), a mutator strain (LT7), and a source of bacteriophage (bacteriophage P22, isolated from the LT22 host strain) that was constructed into the default horizontal gene-transfer tool (P22HT*int*) used for transducing DNA from one *Salmonella* strain to another or backcrossing mutations into wild-type backgrounds to confirm mutation phenotypes. Inasmuch as LT2, LT7, and LT22 have contributed so much to scientific advancement in the last 65 years, the question of whether any of the other strains in the Lilleengen collection have unique characteristics or bacteriophages that could further discoveries in molecular genetics arose. Our laboratory obtained the original LT collection from the Salmonella Genetic Stock Centre (University of Calgary, Calgary, AB, Canada), which had previously received the collection in lyophilized vials from the Lederberg Laboratory, which received it from Kaare Lilleengen (1). We performed microarray assays (2) to determine which *Salmonella* strains in the LT collection were significantly different compared to the published *Salmonella* LT2 sequence (3). Six *Salmonella* strains from the LT collection (LT1, LT18, LT19, LT20, LT21, and LT22) were chosen for next-generation sequencing, followed by analysis of sequence and bacteriophage content.

The chosen LT strains were grown in Luria broth media overnight using a 37°C dry shaking incubator. Total genomic DNA (gDNA) was extracted from 1 mL of each culture using the Pure Link Genomic DNA kit protocol (Invitrogen). Purified gDNA was eluted with 50 µL 10 mM Tris-HCl–0.1 mM EDTA (pH 9.0), and the extracted DNA (80ng/µL) was stored at −20°C. TruSeq DNA PCR-free libraries for each gDNA sample were constructed following protocol (Illumina). Library construction targeted a 350-bp insert. Genomic libraries were combined into a single pool and sequenced in a single-lane 2 × 150 MiSeq run using standard protocols (Illumina version 1.9). We generated an average of 1,027,510 reads measuring 35 to 151 bp per LT strain, approximating 19.3-fold coverage of the reference *Salmonella* genome (LT2) (3). The G+C content ranged from 51 to 52%. Assembly of the whole-genome shotgun data was performed using the SPAdes version 3.10.1 assembler via the CLI version of the PATRIC assembly service (4), filtering for contigs larger than 1,000 bp. Assembly quality was assessed using FastQC version 0.11.2 (5). The RASTtk pipeline (6) was used for annotation as part of the PATRIC annotation service (4).

All of the LT strains sequenced were unique. Each LT strain had two to four intact bacteriophages, two to six incomplete bacteriophages, and one to two questionable bacteriophage regions when submitted to bacteriophage search tool (PHASTER) analysis (7). As expected, LT22 had an intact P22 bacteriophage; notably, LT19, LT20, and LT21 also had an intact P22 bacteriophage, whereas LT1 and LT18 did not. 

### Accession number(s).

This whole-genome shotgun project has been deposited at GenBank under the following accession numbers. LT1, NDGP00000000; LT18, NDGQ00000000; LT19, NDGR00000000; LT20, NDGS00000000; LT21, NDGT00000000; and LT22, NDGU00000000. The versions described in this paper are the first versions, as follows: LT1, NDGP01000000; LT18, NDGQ01000000; LT19, NDGR01000000; LT20, NDGS01000000; LT21, NDGT01000000; and LT22, NDGU01000000.
